# Thiosemicarbazide-Substituted Coumarins as Selective Inhibitors of the Tumor Associated Human Carbonic Anhydrases IX and XII

**DOI:** 10.3390/molecules27144610

**Published:** 2022-07-19

**Authors:** Arzu Gumus, Murat Bozdag, Atilla Akdemir, Andrea Angeli, Silvia Selleri, Fabrizio Carta, Claudiu T. Supuran

**Affiliations:** 1 Department of Chemistry, Faculty of Science and Art, Balikesir University, 10145 Balikesir, Turkey; 2 NEUROFARBA Department, Sezione di Scienze Farmaceutiche e Nutraceutiche, University of Florence, Via Ugo Schiff 6, Sesto Fiorentino, 50019 Florence, Italy; bozdagmurat@gmail.com (M.B.); andrea.angeli@unifi.it (A.A.); silvia.selleri@unifi.it (S.S.); claudiu.supuran@unifi.it (C.T.S.); 3 Computer-Aided Drug Discovery Laboratory, Department of Pharmacology, Faculty of Pharmacy, Bezmialem Vakif University, 34093 Istanbul, Turkey; aakdemir@bezmialem.edu.tr

**Keywords:** carbonic anhydrase inhibitors, privileged scaffolds, coumarins, thiosemicarbazides

## Abstract

A novel series of thiosemicarbazide-substituted coumarins was synthesized and the inhibitory effects against four physiologically relevant carbonic anhydrase isoforms I, II, IX and XII showed selective activities on the tumor-associated IX and XII isozymes. Molecular modeling studies on selected compounds **14a** and **22a** were performed. The binding modes of such compounds were determined assuming their enzymatically active structures (i.e., cinnamic acid) in the thermodynamically favored, and not previously explored, *E* geometry. Molecular modelling suggests multiple interactions within the enzymatic cavity and may explain the high potency and selectivity reported for the hCAs IX and XII.

## 1. Introduction

Extensive original contributions either on coumarin- and thiosemicarbazide-containing structures are reported every year within the field of Medicinal Chemistry and other disciplines not necessarily related to each other [1,2,3,4,5,6,7,8,9]. The unique features of such moieties (i.e., electronic/chemical) along with the straightforward synthetic access are the main reason for them being considered privileged scaffolds endowed with versatile biomedical applications [1,4,7,8,9]. In this context, researchers have largely contributed to the field by demonstrating that the coumarin ring effectively inhibits the α-carbonic anhydrase (CA; EC 4.2.1.1) enzymes and acts as a prodrug by making use of the lactone ring [10]. Such a discovery is of particular value since large amounts of coumarin compounds are derived from natural sources (i.e., in majority from plants) and thus de facto legitimized direct retrieval of CA inhibitors (i.e., CAIs) from the “natural chemical repository”. A variety of biological activities have been attributed to the thiosemicarbazide moiety (i.e., anti-cancer, anti-microbial, anti-viral, antioxidant properties), however a clear definition of its role in biology still remains blurred, mainly regarding the multiple and overlapping biological events triggered [7,8,9]. In this context we sought to investigate the inhibitory effects of coumarin-based CAIs functionalized with variegate thiosemicarbazide-containing tails on the physio/pathologically relevant hCA isoforms I, II, IX and XII. By means of this study we intend to present the first line of knowledge useful to understand the main structural determinants which regulate inhibition potency and selectivity of hCAs.

## 2. Results and Discussion

### 2.1. Synthesis of Coumarins ***14a,b–22a,b*** and ***23b***

**Scheme 1:**
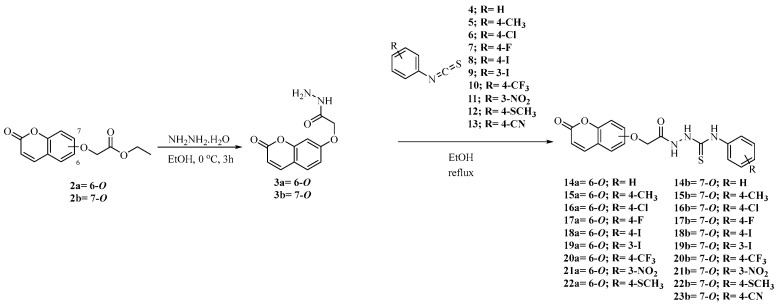
Synthesis of coumarins **14a,b**–**22a,b** and **23b**.

The target compounds were synthesized according to the procedures reported in Scheme 1.

Intermediates **2a,b** and **3a,b** were prepared according to previously reported experimental procedures [6,7] which were subjected to addition reactions on freshly prepared aryl isothiocyanates **4**–**13** [11] to afford the final 6- and 7-substituted coumarins **14a,b**–**22a,b** and **23b**. All final compounds were purified by silica gel column chromatography or crystallization from the appropriate solvents and were fully characterized by means of ^1^H-NMR, ^13^C-NMR and mass spectra.

### 2.2. CA In Vitro Inhibition Assay

The synthesized compounds **14a,b**–**22a,b** and **23b** were investigated in vitro for their inhibition potencies against the four physiologically relevant hCAs I, II, IX and XII, by means of the stopped flow CO_2_ hydrase assay [12]. Commercially available coumarin (**COU**) was used as a reference drug (Table 1).

As expected, and in agreement with the literature data [6], all the compounds were ineffective inhibitors of the house-keeping hCAs I and II with K_i_ values > 10,000 nM. Conversely well-defined structure–activity relationships (SARs) referring to the compound series on the tumor-associated hCAs IX and XII were obtained.

*(i*) As for the isozyme IX and within the 6-substituted coumarin series, the phenyl thioureido derivative **14a** was quite an effective inhibitor, having a K_i_ value of 8.8 nM. Introduction of the tolyl moiety to afford **15a** heavily spoiled the potency up to 26.6-fold (K_i_ of 234 nM). Substitution of the methyl moiety in **15a** with halogens instead (i.e., F, Cl and I) restored the inhibition potencies to a medium-low nanomolar range. Specifically, the 4-chloro derivative **16a** showed a K_i_ value of 48 nM, followed by the 4-fluoro (i.e., K_i_ of **17a** 39 nM) and the 4-iodo which were similar to the unsubstituted derivative **14a** (i.e., K_i_s of 9.6 and 8.8 nM for **18a** and **14a** respectively). Different substitutions at the same position of the phenyl ring, such as the -CF_3_ and the -SCH_3_, heavily affected the K_i_ values which resulted in increases of 9.6- and 5.9-fold for **20a** and **22a**, respectively (i.e., K_i_s of 92 and 57 nM **20a** and **22a**). Shift of the iodo in **18a** to the adjacent 3-position, as in compound **19a**, did not affect the inhibition potency against the hCA IX isozyme (K_i_s of 9.6 nM for either **18a** and **19a**). The presence of the nitro group at 3-position as in compound **21a** determined a K_i_ value of 71 nM. Discrete K_i_ value differences were observed when the 7-coumarin substituted series was compared. For instance, the unsubstituted derivative **14b** was 5.2-fold less effective than its regioisomer **14a** (i.e., K_i_s of 8.8 and 46 nM for **14a** and **14b** respectively). The introduction of the methyl moiety in **14b** to afford **15b** determined a kinetic trend similar to the 6-substituted coumarin series, although only a slight increase of the K_i_ value was reported (i.e., 1.7-fold). Interestingly, the inhibition potency for the 4-substituted halogen derivatives **16b**–**18b** was the opposite when compared to the 6-substituted counterparts **16a**–**18a** (K_i_s of 55, 64 and 721 nM for **16b**, **17b** and **18b**, respectively). No appreciable differences were observed between the regioisomeric pairs **19a** and **19b** (i.e., K_i_s of 9.6 and 9.4 nM for **19a** and **19b** respectively) and the 3-nitro derivative **21b** (K_i_ of 9.1 nM). Close matching differences were observed between the 4-CF_3_ and 4-SCH_3_ pairs, being **20b** and **22b** 1.3- and 1.7-fold more potent than their counterparts **20a** and **22a**, respectively. Finally, the 4-cyano derivative **23b** was the least effective among the entire series with a K_i_ value of 437.5 nM.

(*ii*) Overall, the compounds reported in this study were less effective inhibitors against the second tumor associated hCA XII isoform. The phenyl unsubstituted pairs **14a** and **14b** were equally potent inhibitors, with K_i_s of 739 and 743.5 nM, respectively. Similarly, the tolyl-containing compounds **15a** and **15b** showed very close inhibition potencies (i.e., K_i_s of 709 and 684 nM for **15a** and **15b**, respectively). The halogen-containing derivatives **16a**–**18a** exerted inhibition of the hCA XII at high nanomolar concentrations with kinetic trends identical to the isoform IX (Table 1). Of note is the 4-iodo derivative **18a** which was equally potent on both the tumor-associated isoforms IX/XII (i.e., K_i_s of 9.6 and 8.4 nM for **18a** on hCA IX and XII, respectively). Strong regioisomeric effects were observed when the 4-iodophenyl tail in **18a** was replaced with the 3-iodophenyl moiety to afford **19a** (i.e., K_i_s of 8.4 and 79 nM for **18a** and **19a**, respectively). Replacement of the 3-iodo (**19a**) with a nitro group (**21a**) drastically increased the K_i_ value (i.e., 328.5 nM). The presence at 4-position of the -SCH_3_ group drastically enhanced the inhibition potency for both the 6- and 7-substituted coumarin pairs **22a** and **22b**, which showed K_i_s of 4 and 4.6 nM, respectively. The kinetic trends of **16b**–**18b** were identical to their **16a**–**18a** counterparts, with the chloro derivative **16b** the least effective (K_i_ of 866 nM), followed by the fluoro- and iodo-containing compounds **17b** and **18b** with K_i_s of 745 and 7.5 nM, respectively. In analogy to **18a/19a**, a switch of the halogen to 3-position within the **18b/19b** pair spoiled the inhibition potency against the hCA XII isoforms (i.e., K_i_s of 7.5 and 573 nM for **18b** and **19b** respectively). Interestingly, the 4-CF_3_-phenyl derivatives **20a** and **20b** showed a kinetic trend on the hCA XII comparable to hCA IX isozyme. Enhanced regioisomeric effects were observed for the hCA XII, with **20b** and **21b** up to 5.4- and 38.2-fold more effective than **20a** and **21a**, respectively. Finally, also for the hCA XII isozyme the 4-CN derivative **23b** was a high nanomolar inhibitor (i.e., K_i_ of 473 nM).

### 2.3. Molecular Modelling Studies

**Figure 1:**
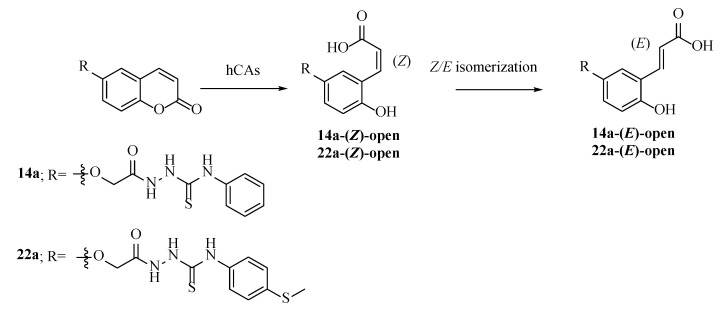
Hydrolysis of the coumarin scaffolds and *Z*/*E* geometrical isomerization.

We performed molecular modelling studies in order to decipher the molecular features underlying the in vitro K_i_ value differences occurring among the compounds set. Specifically, we turned our attention to **14a**, which was the strongest hCA IX inhibitor within the series (K_i_ of 8.8 nM) and was 84-fold more potent than the hCA XII (K_i_ of 739 nM). In contrast, compound **22a** was highly effective on the hCA XII (K_i_ of 4.1 nM) and 14-fold less effective on the IX isoform (K_i_ of 57 nM). Considering the mechanism of action of the coumarin moiety towards CAs [10], we assumed the coumarin warheads in either **14a** and **22a** were the open/hydrolyzed form which underwent f an isomerization to the thermodynamically more stable *E* isomer (i.e., hereafter referred as **14a-(*E*)-open** and **22a-(*E*)-open**, Figure 1) and thus docked into the hCA IX and XII crystal structure cavity sites.

The obtained ligand–enzyme complexes were subjected to 250 ns molecular dynamics (MD) simulations which confirmed the interaction of ligands within the hCA IX/XII isoforms.

#### Modelling Studies of hCA IX– Hydrolyzed **14a**-Open

**Figure 2:**
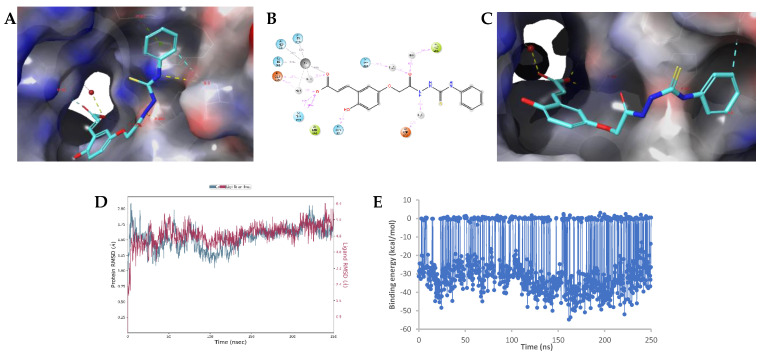
(**A**) The docked pose **14a-(*E*)-open**. (**B**) Ligand–protein binding interactions observed for 30% or more of the duration of the unrestricted MD simulation. (**C**) Ligand pose after a 250 MD simulation. (**D**) RMSD value of the protein Cα-atoms and the ligand heavy atoms. (**E**) MM-GBSA binding energy. In the 2D plots, anionic residues are indicated in red, cationic residues are indicated in purple, hydrophobic residues and surfaces are indicated in green and hydrophilic residues and surfaces are indicated in light blue. Hydrogen bonds are indicated in purple (2D plots) or yellow (**A**,**C**) dashed lines.

Simulation on the hCA IX in complex with **14a-(*E*)-open** indicated stable interactions occurring between the ligand carboxylic moiety to the zinc-bound water molecule and by means of a second water molecule to Thr199 and to the Zn^2+^ ion (Figure 2). During MD simulations both waters could interact with the Zn^2+^ as well as Glu106. A direct hydrogen bond was observed with Gln92 (40%) and water-mediated hydrogen interactions were observed with Asp132 (31%), Thr200 (36%) and Pro201 (46%). The RMSD values for the protein revealed a stable protein structure, while the ligand was observed to be more dynamic, which is mainly due to the movement of the terminal phenyl group (Figure 2A,C). The MM-GBSA binding energy fluctuated between approximately −55 and 3 kcal/mol during the simulation with an average of −24.19 kcal/mol and a standard deviation of 16.1 kcal/mol.

**Figure 3:**
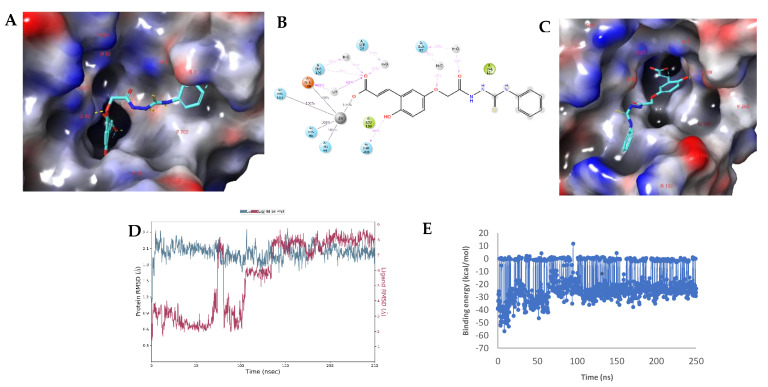
Modelling results of the hCA IX–**14a-(*E*)-open** interacting with Zn^2+^ ion. (**A**) Docked pose compound **14a-(*E*)-open**. (**B**) Ligand–protein binding interactions observed for 30% or more of the duration of the unrestricted MD simulation. (**C**) Ligand pose after a 250 MD simulation. (**D**) RMSD value of the protein Cα-atoms and the ligand heavy atoms. (**E**) MM-GBSA binding energy. In the 2D plots, anionic residues are indicated in red, cationic residues are indicated in purple, hydrophobic residues and surfaces are indicated in green and hydrophilic residues and surfaces are indicated in light blue. Hydrogen bonds are indicated in purple (2D plots) or yellow (**A**,**C**) dashed lines.

Docking studies indicated that compound **14a-(*E*)-open** may also interact directly with the hCA IX metal ion. A hydrogen bond with the backbone NH group of Thr199 (Figure 3) was also formed. In addition, hydrogen bonds were observed with the side chain of Gln92 and the backbone of Pro201. During a 250 ns MD simulation, the RMSD values of the protein Cα atoms were in the 1.8–2.4 Å range, while the ligand was much higher to reach approximately 8 Å. Major ligand–protein interactions during the simulation occurred with Zn^2+^ (100%), Thr199 (35%) and Thr200 (49%). Many hydrogen bonds via bridging water molecules as well as hydrophobic interactions were also observed. The snapshot at 250 ns clearly shows that **14a-(*E*)-open** adopted a different orientation within the active site, while the interaction with the metal remained. The MM-GBSA binding energy fluctuated between approximately −57 and 12 kcal/mol during the simulation with an average of −20.28 kcal/mol and a standard deviation of 12.97 kcal/mol.

### 2.4. Modelling Studies of hCA XII–Hydrolyzed ***22a***-Open

**Figure 4:**
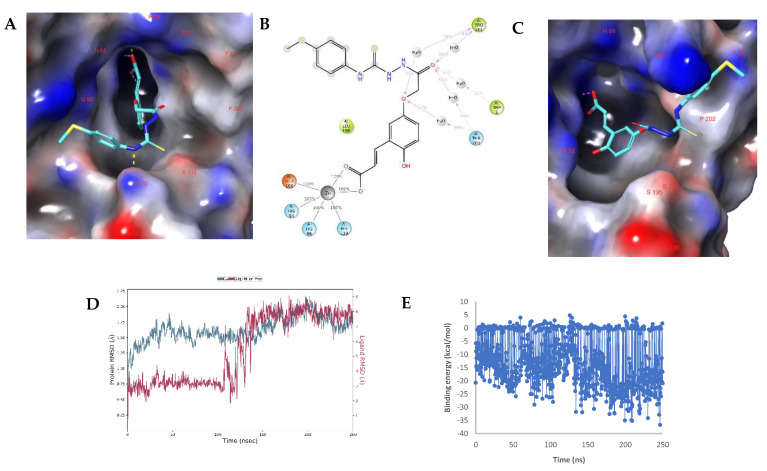
Molecular modelling results of the hCA XII–**22a-(*E*)-open** interaction. (**A**) Docked pose of **22a-(*E*)-open**. (**B**) The ligand–protein binding interactions observed for 30% or more of the duration of the unrestricted MD simulation. (**C**) Ligand pose after a 250 MD simulation. (**D**) RMSD value of the protein Cα-atoms and the ligand heavy atoms. (**E**) Binding energy. In the 2D plots, anionic residues are indicated in red, cationic residues are indicated in purple, hydrophobic residues and surfaces are indicated in green and hydrophilic residues and surfaces are indicated in light blue. Hydrogen bonds are indicated in purple (2D plots) or yellow (**A**,**C**) dashed lines.

**Figure 5:**
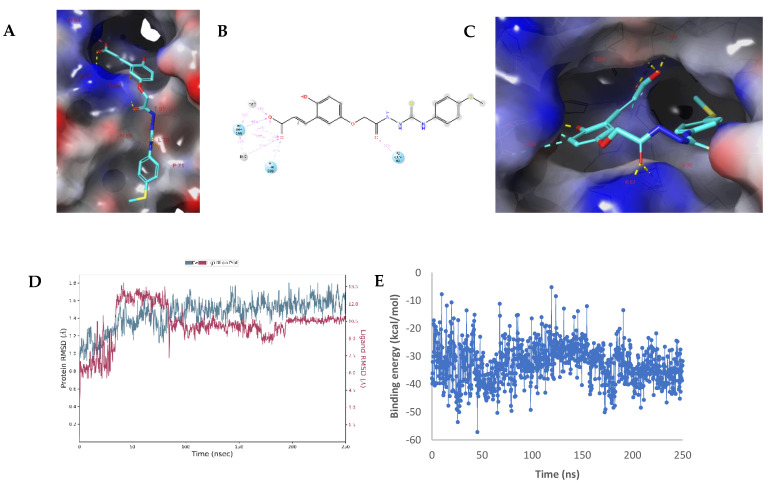
Molecular modelling results of the hCA XII–**22a-(*E*)-open**. (**A**) Docked pose compound **22a-(*E*)-open**. (**B**) Ligand–protein binding interactions observed for 30% or more of the duration of the unrestricted MD simulation. (**C**) Ligand pose after a 250 MD simulation. (**D**) RMSD value of the protein Cα-atoms and the ligand heavy atoms. (**E**) Binding energy. In the 2D plots, anionic residues are indicated in red, cationic residues are indicated in purple, hydrophobic residues and surfaces are indicated in green and hydrophilic residues and surfaces are indicated in light blue. Hydrogen bonds are indicated in purple (2D plots) or yellow (**A**,**C**) dashed lines.

To our surprise, the **22a-(*E*)-open** revealed interactions within the hCA XII active site either with the metal ion (Figure 4) or the active site entrance amino acids (Figure 5). A pose in which the substrate carboxylic moiety interacted with the zinc-bound water was obtained, but was unstable during a 250 ns MD simulation.

As above, **22a-(*E*)-open** interacted with the catalytic Zn^2+^ of the hCA XII during the entire duration of the MD simulation (Figure 5). The docked pose also revealed a hydrogen bond interaction that was established with the side chain of Ser132, which however was not stable during simulations as the ligand adopted different orientations within the enzymatic site to form water-bridged hydrogen bonds as well as hydrophobic interactions. The MM-GBSA binding energy fluctuated between approximately −36 and 5 kcal/mol during the simulation with an average of −12.76 kcal/mol and a standard deviation of 9.44 kcal/mol. As depicted in Figure 5, **22a-(*E*)-open** also interacted with the entrance amino acids of the hCA XII active cleft. In the docked pose, the carboxylic acid moiety was hydrogen bonded with His64 and Lys67 and additional interactions were with Leu70, Thr91 and Gln92 (Figure 5). During the MD simulation the ligand’s RMSD increased towards approximately 10 Å, while the protein Cα RMSD values remained low. This indicated large re-orientation of the ligand. Specifically, the carboxylic acid moved away from His64 and Lys67 and formed water-mediated and direct hydrogen bonds with Thr199 and direct hydrogen bonds with Thr200 instead. In addition, a hydrogen bond was formed between the ligand carbonyl group and Gln92. The MM-GBSA binding energy fluctuated between approximately −57.2 and 5.2 kcal/mol during the simulation with an average of −32.8 kcal/mol and a standard deviation of 7.0 kcal/mol.

Overall, the isoform selectivity of **14** and **22** on hCA IX and XII, respectively, was assessed by means of modelling studies applied to their enzymatic active forms **14a-(*E*)-open** and **22a-(*E*)-open**. The obtained data indicated that **14a-(*E*)-open** was able to interact within the hCA IX active site by means of its carboxylic acid to the zinc-bound water molecule (−24.19 ± 16.10 kcal/mol) or directly to the Zn^2+^ ion (−20.28 ± 12.97 kcal/mol). As for hCA XII-**22a-(*E*)-open**, the main interactions detected were between the carboxylic moiety and the Zn^2+^ ion (−12.76 ± 9.44 kcal/mol) or the entrance amino acids (−32.80 ± 7.0 kcal/mol). The different methods of the ligands in targeting hCA’s active sites and the dynamic bindings make it difficult to directly relate the calculated MM-GBSA binding energy to the measured K_i_ values, which are also likely dependent on the free energy changes resulting from displacing water molecules within the active sites [13,14,15,16].

## 3. Materials and Methods

### 3.1. Chemistry

All anhydrous solvents and reagents used in this study were purchased from Alfa Aesar, TCI, and Sigma-Aldrich. The synthetic reactions involving air- or moisture-sensitive chemicals were carried out under a nitrogen atmosphere using dried glassware and syringe techniques in order to transfer the solutions. Melting points were determined in open capillaries in an electrical melting point apparatus and are uncorrected. Nuclear magnetic resonance (^1^H-, ^13^C-, and ^19^F-NMR) spectra were recorded using a Bruker Avance III 400 MHz spectrometer using DMSO-*d*_6_ as solvent. The chemical shifts are reported in parts per million (ppm), and the coupling constants (*J*) are expressed in Hertz (Hz). The splitting patterns are designated as s, singlet; d, doublet; t, triplet; q, quartet; m, multiplet; brs, broad singlet; dd, doublet of doublets. The correct assignment of exchangeable protons (i.e., O*H* and N*H*) was carried out by means of the addition of D_2_O. The high-resolution mass spectrometry (HRMS) analysis was performed with a Thermo Finnigan LTQ Orbitrap mass spectrometer coupled with an electrospray ionization source (ESI).

### 3.2. General Procedure for Synthesis of ***14a,b**–**22a,b*** and ***23b***

A solution of coumarin **3a**–**b** (0.1g, 0.43 mmol, 1.0 equv) in EtOH (20 mL) was treated with phenylisothiocyanate **4–13** (0.43 mmol, 1.0 equv) then the reaction was refluxed overnight (ON). The reaction mixture was cooled to room temperature and the formed precipitate was filtered-off, washed with Et_2_O (3 × 5 mL) dried under vacuum to obtain a residue which was recrystallized from EtOH/H_2_O to afford the desired products **14a,b**–**22a,b** and **23b** as solids.

*N-Phenyl-2-(2-((2-oxo-2H-chromen-6-yl)oxy)acetyl)hydrazine-1-carbothioamide* **14a**. 76% yield; m.p. 173–175 °C; ^1^H-NMR (400 MHz, DMSO-*d*_6_) δ 4.72 (2H, s), 6.54 (1H, d, *J* 9.6), 7.21 (1H, t, *J* 7.1), 7.37 (7H, m), 8.02 (1H, d, *J* 9.6), 9.71 (2H, m, exchange with D_2_O, NHCSNH), 10.37 (1H, s, exchange with D_2_O, NHCO); ^13^C-NMR (100 MHz, DMSO-*d*_6_) δ 67.6, 112.7, 117.6, 118.3, 120.0, 120.9, 126.0, 129.0, 129.8, 139.9, 144.8, 149.1, 154.9, 160.9, 168.1, 181.9; *m*/*z* calcd. for C_18_H_15_N_3_O_4_S (369.4), found: (ESI negative) 368.0 [M − H]^−^; ESI-HRMS (*m*/*z*) calculated for [M + H]^+^ ion species C_18_H_16_N_3_O_4_S = 370.0783, found 370.0780.

*N-Phenyl-2-(2-((2-oxo-2H-chromen-7-yl)oxy)acetyl)hydrazine-1-carbothioamide* **14b**. 80% yield; m.p. 155–156 °C; ^1^H-NMR (400 MHz, DMSO-*d*_6_) δ 4.81 (2H, s), 6.35 (1H, d, *J* 9.5), 7.08 (2H, m), 7.21 (1H, t, *J* 7.6), 7.38 (2H, t, *J* 7.6), 7.47 (2H, d, *J* 7.6), 7.70 (1H, d, *J* 8.5), 8.04 (1H, d, *J* 9.5), 9.71 (2H, m, exchange with D_2_O, NHCSNH), 10.37(1H, s, exchange with D_2_O, NHCO); ^13^C-NMR (100 MHz, DMSO-*d*_6_) δ 67.3, 102.5, 113.7, 113.8, 118.9, 126.1, 126.8, 129.0, 130.4, 139.9, 145.1, 156.1, 161.1, 161.7, 167.8, 181.9; *m*/*z* calcd. for C_18_H_15_N_3_O_4_S (369.4), found: (ESI negative) 368.0 [M − H]^−^; ESI-HRMS (*m*/*z*) calculated for [M + H]^+^ ion species C_18_H_16_N_3_O_4_S = 370.0783, found 370.0784. Experimental values in agreement with reported data [7].

*N-(p-Tolyl)-2-(2-((2-oxo-2H-chromen-6-yl)oxy)acetyl)hydrazine-1-carbothioamide* **15a**. 73% yield; m.p. 167–169 °C; ^1^H-NMR (400 MHz, DMSO-*d*_6_) δ 2.32 (3H, s), 4.72 (2H, s), 6.54 (1H, d, *J* 9.6), 7.17 (2H, d, *J* 8), 7.35 (5H, m), 8.01 (1H, d, *J* 9.6), 9.64 (2H, m, exchange with D_2_O, NHCSNH), 10.34 (1H, s, exchange with D_2_O, NHCO); ^13^C-NMR (100 MHz, DMSO-*d*_6_) δ 21.6, 67.7, 112.9, 117.7, 118.5, 120.1, 121.2, 129.6, 130.4, 135.5, 137.4, 145.0, 149.3, 155.1, 161.2, 168.4, 182.4; *m*/*z* calcd. for C_19_H_17_N_3_O_4_S (383.4), found: (ESI negative) 382.0 [M − H]^−^; ESI-HRMS (*m*/*z*) calculated for [M + H]^+^ ion species C_19_H_18_N_3_O_4_S = 384.0940, found 384.0942.

*N-(p-Tolyl)-2-(2-((2-oxo-2H-chromen-7-yl)oxy)acetyl)hydrazine-1-carbothioamide* **15b**. 80% yield; m.p. 147–149 °C (dec); ^1^H-NMR (400 MHz, DMSO-*d*_6_) δ 2.32 (3H, s), 4.80 (2H, s), 6.35 (1H, d, *J* 9.5), 7.08 (2H, m), 7.18 (2H, d, *J* 8.0), 7.33 (2H, d, *J* 8.0), 7.69 (1H, d, *J* 8.4), 8.04 (1H, d, *J* 9.5), 9.63 (1H, s, exchange with D_2_O, NHCSNH), 9.66 (1H, s, exchange with D_2_O, NHCSNH), 10.32 (1H, s, exchange with D_2_O, NHCO); ^13^C-NMR (100 MHz, DMSO-*d*_6_) δ 21.4, 67.3, 102.5, 113.7, 113.7, 113.8, 126.7, 129.5, 130.4, 135.3, 137.3, 145.1, 156.1, 161.1, 161.7, 167.8, 182.0; *m*/*z* calcd. for C_19_H_17_N_3_O_4_S (383.4), found: (ESI negative) 382.0 [M − H]^−^; ESI-HRMS (*m/z*) calculated for [M + H]+ ion species C_19_H_18_N_3_O_4_S = 384.0940, found 384.0943.

*N-(4-Chlorophenyl)-2-(2-((2-oxo-2H-chromen-6-yl)oxy)acetyl)hydrazine-1-carbothioamide* **16a**. 64% yield; m.p. 163–165 °C; ^1^H-NMR (400 MHz, DMSO-*d*_6_) δ 4.73 (2H, s), 6.54 (1H, d, *J* 9.6), 7.35 (2H, m), 7.43 (3H, m), 7.50 (2H, d, *J* 8.0), 8.02 (1H, d, *J* 9.6), 9.81 (2H, m, exchange with D_2_O, NHCSNH), 10.38 (1H, s, exchange with D_2_O, NHCO); ^13^C-NMR (100 MHz, DMSO-*d*_6_) δ 67.6, 112.7, 117.6, 118.3, 120.0, 120.9, 128.9, 129.5, 132.3, 138.9, 144.8, 149.1, 154.9, 160.9, 168.2, 182.0; *m*/*z* calcd. for C_18_H_14_ClN_3_O_4_S (403.8), found: (ESI negative) 402.0 [M − H]^−^; ESI-HRMS (*m/z*) calculated for [M + H]^+^ ion species C_18_H_15_ClN_3_O_4_S = 404.0394, found 404.0397.

*N-(4-Chlorophenyl)-2-(2-((2-oxo-2H-chromen-7-yl)oxy)acetyl)hydrazine-1-carbothioamide* **16b.** 67% yield; m.p. 202–203 °C; ^1^H-NMR (400 MHz, DMSO-*d*_6_) δ 4.81 (2H, s), 6.35 (1H, d, *J* 9.5), 7.08 (2H, m), 7.44 (2H, d, *J* 8.4), 7.52 (2H, d, *J* 8.4), 7.70 (1H, d, *J* 8.5), 8.04 (1H, d, *J* 9.5), 9.82 (2H, m, exchange with D_2_O, NHCSNH), 10.39 (1H, s, exchange with D_2_O, NHCO); ^13^C-NMR (100 MHz, DMSO-*d*_6_) δ 67.2, 102.5, 113.7, 113.8, 114.0, 119.9, 128.9, 129.8, 130.3, 138.9, 145.1, 156.1, 161.1, 161.6, 167.8, 182.0; *m*/*z* calcd. for C_18_H_14_ClN_3_O_4_S (403.8), found: (ESI negative) 402.0 [M − H]^−^; ESI-HRMS (*m/z*) calculated for [M + H]^+^ ion species C_18_H_15_ClN_3_O_4_S = 404.0394, found 404.0399. Experimental values in agreement with reported data [17].

*N-(4-Fluorophenyl)-2-(2-((2-oxo-2H-chromen-6-yl)oxy)acetyl)hydrazine-1-carbothioamide* **17a**. 60% yield; m.p. 181–183 °C; ^1^H-NMR (400 MHz, DMSO-*d*_6_) δ 4.72 (2H, s), 6.54 (1H, d, *J* 9.6), 7.21 (2H, m), 7.33–7.45 (5H, m), 8.02 (1H, d, *J* 9.6), 9.73 (2H, m, exchange with D_2_O, NHCSNH), 10.37(1H, s, exchange with D_2_O, NHCO); ^13^C-NMR (100 MHz, DMSO-*d*_6_) δ 67.6, 112.8, 115.7 (d, ^2^*J* 22.4), 117.6, 118.3, 120.0, 121.0, 128.9 (m), 136.3 (d, ^4^*J* 3), 144.8, 149.1, 155.0, 160.5 (d, ^1^*J* 239.7), 161.0, 168.17, 182.3; ^19^F-NMR (376 MHz, DMSO-*d*_6_) δ −117.1 (1F, s); *m*/*z* calcd. for C_18_H_14_FN_3_O_4_S (387.4), found: (ESI negative) 386.0 [M − H]^−^; ESI-HRMS (*m/z*) calculated for [M + H]^+^ ion species C_18_H_15_FN_3_O_4_S = 388.0689, found 388.0685.

*N-(4-Fluorophenyl)-2-(2-((2-oxo-2H-chromen-7-yl)oxy)acetyl)hydrazine-1-carbothioamide* 1**7b**. 85% yield; m.p. 119–121 °C (dec); ^1^H-NMR (400 MHz, DMSO-*d*_6_) δ 4.81 (2H, s), 6.35 (1H, d, *J* 9.5), 7.06–7.10 (2H, m), 7.21 (2H, m), 7.46 (2H, m), 7.70 (1H, d, *J* 8.4), 8.04 (1H, d, *J* 9.5), 9.74 (2H, m, exchange with D_2_O, NHCSNH), 10.36 (1H, s, exchange with D_2_O, NHCO); ^13^C-NMR (100 MHz, DMSO-*d*_6_) δ 67.6, 102.5, 113.7, 113.8, 113.8, 115.7 (d, ^2^*J* 22.4), 128.9 (d, ^3^*J* 9), 130.4, 136.2 (d, ^4^*J* 3), 145.2, 156.1, 160.5 (d, ^1^*J* 240.5) 161.2, 161.7, 167.8, 182.3; ^19^F-NMR (376 MHz, DMSO-*d*_6_) δ −117.1 (1F, s); *m*/*z* calcd. for C_18_H_14_FN_3_O_4_S (387.4), found: (ESI negative) 386.0 [M − H]^−^; ESI-HRMS (*m/z*) calculated for [M + H]^+^ ion species C_18_H_15_FN_3_O_4_S = 388.0689, found 388.0687. Experimental values in agreement with reported data [17].

*N-(4-Iodophenyl)-2-(2-((2-oxo-2H-chromen-6-yl)oxy)acetyl)hydrazine-1-carbothioamide* **18a.** 79% yield; m.p. 160–162 °C; ^1^H-NMR (400 MHz, DMSO-*d*_6_) δ 4.72 (2H, s), 6.54 (1H, d, *J* 9.6), 7.34 (4H, m), 7.42 (1H, d, *J* 8.9), 7.72(2H, d, *J* 8.4), 8.02 (1H, d, *J* 9.6), 9.71 (1H, s, exchange with D_2_O, NHCSNH), 9.80 (1H, s, exchange with D_2_O, NHCSNH), 10.37 (1H, s, exchange with D_2_O, NHCO); ^13^C-NMR (100 MHz, DMSO-*d*_6_) δ 67.6, 90.7, 112.7, 117.6, 118.3, 120.0, 120.9, 128.8, 137.7, 139.8, 144.8, 149.1, 154.9, 160.9, 168.1, 181.8; *m*/*z* calcd. for C_18_H_14_IN_3_O_4_S (495.3), found: (ESI negative) 494.0 [M − H]^−^; ESI-HRMS (*m*/*z*) calculated for [M + H]^+^ ion species C_18_H_15_IN_3_O_4_S = 494.9750, found 494.9753.

*N-(4-Iodophenyl)-2-(2-((2-oxo-2H-chromen-7-yl)oxy)acetyl)hydrazine-1-carbothioamide* **18b**. 90% yield; m.p. 164–166 °C; ^1^H-NMR (400 MHz, DMSO-*d*_6_) δ 4.81 (2H, s), 6.35 (1H, d, *J* 9.5), 7.08 (2H, m), 7.34 (2H, d, *J* 8.2), 7.71 (3H, m), 8.04 (1H, d, *J* 9.5), 9.81 (2H, m, exchange with D_2_O, NHCSNH), 10.37 (1H, s, exchange with D_2_O, NHCO); ^13^C-NMR (100 MHz, DMSO-*d*_6_) δ 67.2, 90.8, 102.5, 113.7, 113.7, 128.7, 130.1, 130.3, 137.7, 139.8, 145.1, 156.0, 161.0, 161.6, 167.7, 181.8; *m*/*z* calcd. for C_18_H_14_IN_3_O_4_S (495.3), found: (ESI negative) 494.0 [M − H]^−^; ESI-HRMS (*m*/*z*) calculated for [M + H]^+^ ion species C_18_H_15_IN_3_O_4_S = 494.9750, found 494.9748.

*N-(3-Iodophenyl)-2-(2-((2-oxo-2H-chromen-6-yl)oxy)acetyl)hydrazine-1-carbothioamide* **19a**. 80% yield; m.p. 144–146 °C; ^1^H-NMR (400 MHz, DMSO-*d*_6_) δ 4.73 (2H, s), 6.54 (1H, d, *J* 9.6), 7.18 (1H, t, *J* 8), 7.34 (1H, dd, *J* 8.9, 2.7), 7.37 (1H, m), 7.42 (1H, d, *J* 8.9), 7.56 (2H, m), 7.85 (1H, s), 8.02 (1H, d, *J* 9.6), 9.74(1H, s, exchange with D_2_O, NHCSNH), 9.85 (1H, s, exchange with D_2_O, NHCSNH), 10.38 (1H, s, exchange with D_2_O, NHCO); ^13^C-NMR (100 MHz, DMSO-*d*_6_) δ 67.6, 94.1, 112.7, 117.6, 118.3, 120.0, 120.9, 125.9, 130.9, 134.5, 141.4, 144.7, 149.1, 154.9, 160.9, 168.2, 181.7; *m*/*z* calcd. for C_18_H_14_IN_3_O_4_S (495.3), found: (ESI negative) 494.0 [M − H]^−^; ESI-HRMS (*m/z*) calculated for [M + H]^+^ ion species C_18_H_15_IN_3_O_4_S = 494.9750, found 494.9752.

*N-(3-Iodophenyl)-2-(2-((2-oxo-2H-chromen-7-yl)oxy)acetyl)hydrazine-1-carbothioamide* **19b**. 88% yield; m.p. 168–169 °C; ^1^H-NMR (400 MHz, DMSO-*d*_6_) δ 4.80 (2H, s), 6.35 (1H, d, *J* 9.5), 7.07 (2H, m), 7.19 (1H, t, *J* 8), 7.56 (2H, d, *J* 8), 7.70 (1H, d, *J* 9), 7.87 (1H, s), 8.04 (1H, d, *J* 9.5), 9.76 (1H, s, exchange with D_2_O, NHCSNH), 9.86 (1H, s, exchange with D_2_O, NHCSNH) 10.39 (1H, s, exchange with D_2_O, NHCO); ^13^C-NMR (100 MHz, DMSO-*d*_6_) δ 67.3, 94.1, 102.5, 113.7, 113.7, 113.8, 126.0, 130.3, 130.9, 134.5, 141.3, 145.1, 156.0, 161.0, 161.6, 167.8, 181.7; *m*/*z* calcd. for C_18_H_14_IN_3_O_4_S (495.3), found: (ESI negative) 494.0 [M − H]^−^; ESI-HRMS (*m/z*) calculated for [M + H]^+^ ion species C_18_H_15_IN_3_O_4_S = 494.9750, found 494.9757.

*N-(4-(Trifluoromethyl)phenyl)-2-(2-((2-oxo-2H-chromen-6-yl)oxy)acetyl)hydrazine-1-carbothioamide***20a.** 71% yield; m.p. 182–184 °C; ^1^H-NMR (400 MHz, DMSO-*d*_6_) δ 4.74 (2H, s), 6.54 (1H, d, *J* 9.6), 7.33–7.38 (2H, m), 7.42 (1H, d, *J* 9), 7.75 (4H, m), 8.02 (1H, d, *J* 9.6), 9.96 (2H, m, exchange with D_2_O, NHCSNH), 10.43 (1H, s, exchange with D_2_O, NHCO); ^13^C-NMR (100 MHz, DMSO-*d*_6_) δ 67.7, 112.8, 117.6, 118.3, 120.0, 121.0, 125.2 (q, ^1^*J* 268), 126.1 (q, ^2^*J* 32), 143.8, 144.8, 149.2, 154.9, 161.0, 168.4, 182.1; ^19^F-NMR (376 MHz, DMSO-*d*_6_) δ − 60.4 (3F, s); *m*/*z* calcd. for C_19_H_14_F_3_N_3_O_4_S (437.4), found: (ESI negative) 436.0 [M − H]^−^; ESI-HRMS (*m/z*) calculated for [M + H]^+^ ion species C_18_H_15_F_3_N_3_O_4_S = 438.0657, found 437.0653.

*N-(4-(Trifluoromethyl)phenyl)-2-(2-((2-oxo-2H-chromen-7-yl)oxy)acetyl)hydrazine-1-carbothioamide***20b**. 81% yield; m.p. 207–209 °C; ^1^H-NMR (400 MHz, DMSO-*d*_6_) δ 4.83 (2H, s), 6.36 (1H, d, *J* 9.5), 7.07–7.12 (2H, m), 7.70 (1H, d, *J* 8.6), 7.74 (2H, m), 7.81 (2H, m), 8.04 (1H, d, *J* 9.5), 9.96 (2H, m, exchange with D_2_O, NHCSNH), 10.43 (1H, s, exchange with D_2_O, NHCO); ^13^C-NMR (100 MHz, DMSO-*d*_6_) δ 67.2, 102.5, 113.7, 113.8, 125.2 (q, ^1^*J* 270), 126.1 (q, ^2^*J* 32), 126.6 (q, ^3^*J* 4), 130.4, 143.7 (1, ^4^*J* 1), 145.1, 156.1, 161.1, 161.7, 168.0, 181.9; ^19^F-NMR (376 MHz, DMSO-*d*_6_) δ − 60.4 (3F, s); *m*/*z* calcd. for C_19_H_14_F_3_N_3_O_4_S (437.4), found: (ESI negative) 436.0 [M − H]^−^; ESI-HRMS (*m/z*) calculated for [M + H]^+^ ion species C_18_H_15_F_3_N_3_O_4_S = 438.0657, found 437.0652.

*N-(3-Nitrophenyl)-2-(2-((2-oxo-2H-chromen-6-yl)oxy)acetyl)hydrazine-1-carbothioamide* **21a**. 69% yield; m.p. 210–212 °C; ^1^H-NMR (400 MHz, DMSO-*d*_6_) δ 4.75 (2H, s), 6.54 (1H, d, *J* 9.6), 7.39 (3H, m), 7.67 (1H, t, *J* 8.2), 8.03 (3H, m), 8.47 (1H, s), 10.04 (2H, m, exchange with D_2_O, NHCSNH), 10.46 (1H, s, exchange with D_2_O, NHCO); ^13^C-NMR (100 MHz, DMSO-*d*_6_) δ 67.6, 112.7, 117.6, 118.3, 120.0, 120.5, 120.9, 130.2, 132.5, 141.2, 144.8, 148.1, 149.1, 154.8, 160.9, 168.3, 181.9; *m*/*z* calcd. for C_14_H_14_N_4_O_6_S (414.4), found: (ESI negative) 413.0 [M − H]^−^; ESI-HRMS (*m/z*) calculated for [M + H]^+^ ion species C_14_H_15_N_4_O_6_S = 415.0634, found 415.0631.

*N-(3-Nitrophenyl)-2-(2-((2-oxo-2H-chromen-7-yl)oxy)acetyl)hydrazine-1-carbothioamide* **21b**. 62% yield; m.p. 214–215 °C; ^1^H-NMR (400 MHz, DMSO-*d*_6_) δ 4.83 (2H, s), 6.36 (1H, d, *J* 9.5), 7.09 (2H, m), 7.68 (2H, m), 8.04 (3H, m), 8.51 (1H, s), 10.06 (2H, m, exchange with D_2_O, NHCSNH), 10.46 (1H, s, exchange with D_2_O, NHCO); ^13^C-NMR (100 MHz, DMSO-*d*_6_) δ 67.3, 102.5, 113.8, 113.8, 120.6, 129.6, 130.3, 130.4, 132.4, 132.6, 141.2, 145.2, 148.2, 156.1, 161.1, 161.6, 168.1, 181.9; *m*/*z* calcd. for C_14_H_14_N_4_O_6_S (414.4), found: (ESI negative) 413.0 [M − H]^−^; ESI-HRMS (*m/z*) calculated for [M + H]^+^ ion species C_14_H_15_N_4_O_6_S = 415.0634, found 415.0637.

*N-(4-(Methylthio)phenyl)-2-(2-((2-oxo-2H-chromen-6-yl)oxy)acetyl)hydrazine-1-carbothioamide* **22a**. 82% yield; m.p. 148–150 °C, ^1^H-NMR (400 MHz, DMSO-*d*_6_) δ 2.57 (3H, s), 4.75 (2H,s), 6.57 (1H, d, *J* 9.4), 7.30 (2H, d, *J* 7.8), 7.41 (5H, m), 8.05 (1H, d, *J* 9.4), 9.73 (2H, m, exchange with D_2_O, NHCSNH), 10.38 (1H, s, exchange with D_2_O, NHCO); ^13^C-NMR (100 MHz, DMSO-*d*_6_) δ 16.0, 67.6, 112.7, 117.6, 118.2, 120.0, 120.9, 126.7, 135.3, 137.1, 144.8, 149.1, 154.9, 160.9, 168.1, 181.9; *m*/*z* calcd. for C_19_H_17_N_3_O_4_S_2_ (415.5), found: (ESI negative) 414.0 [M − H]^−^; ESI-HRMS (*m/z*) calculated for [M + H]^+^ ion species C_14_H_18_N_3_O_4_S_2_ = 416.0660, found 416.0658.

*N-(4-(Methylthio)phenyl)-2-(2-((2-oxo-2H-chromen-7-yl)oxy)acetyl)hydrazine-1-carbothioamide* **22b**. 94% yield; m.p. 163–164 °C; ^1^H-NMR (400 MHz, DMSO-*d*_6_) δ 2.50 (3H, s), 4.81 (2H, s), 6.35 (1H, d, *J* 9.5), 7.08 (2H, m), 7.28 (2H, d, *J* 8.6), 7.42 (2H, d, *J* 8.6), 7.70 (1H, d, *J* 8.5), 8.04 (1H, d, *J* 9.5), 9.71 (2H, m, exchange with D_2_O, NHCSNH), 10.35 (1H, s, exchange with D_2_O, NHCO); ^13^C-NMR (100 MHz, DMSO-*d*_6_) δ 16.0, 67.3, 102.6, 113.8, 113.8, 113.9, 126.8, 129.6, 130.4, 132.5, 137.1, 145.2, 156.1, 161.2, 161.7, 167.9, 182.2; *m*/*z* calcd. for C_19_H_17_N_3_O_4_S_2_ (415.5), found: (ESI negative) 414.0 [M − H]^−^; ESI-HRMS (*m/z*) calculated for [M + H]^+^ ion species C_14_H_18_N_3_O_4_S_2_ = 416.0660, found 416.0663.

*N-(4-Cyanophenyl)-2-(2-((2-oxo-2H-chromen-7-yl)oxy)acetyl)hydrazine-1-carbothioamide* **23b**. 80% yield; m.p. 224–226 °C; ^1^H-NMR (400 MHz, DMSO-*d*_6_) δ 4.83 (2H, s), 6.36 (1H, d, *J* 9.5), 7.08 (2H, m), 7.70 (1H, d, *J* 8.5), 7.84 (4H, m), 8.04 (1H, d, *J* 9.5), 10.04 (2H, m, exchange with D_2_O, NHCSNH), 10.45 (1H, s, exchange with D_2_O, NHCO); ^13^C-NMR (100 MHz, DMSO-*d*_6_) δ 67.3, 102.3, 102.5, 107.8, 113.8, 113.8, 119.8, 126.3, 130.4, 133.2, 144.5, 145.2, 156.1, 161.1, 161.6, 168.0, 181.6; *m*/*z* calcd. for C_19_H_14_N_4_O_4_S (394.4), found: (ESI negative) 393.0 [M − H]^−^; ESI-HRMS (*m/z*) calculated for [M + H]^+^ ion species C_19_H_15_N_4_O_4_S = 395.0736, found 395.0738.

### 3.3. In Vitro Carbonic Anhydrase Inhibition

An Applied Photophysics stopped-flow instrument was used to assay the CA catalyzed CO_2_ hydration activity [12]. Phenol red (at a concentration of 0.2 mM) was used as an indicator, working at the absorbance maximum of 557 nm, with 20 mM Hepes (pH 7.4) as a buffer, and 20 mM Na_2_SO_4_ (to maintain constant ionic strength), following the initial rates of the CA-catalyzed CO_2_ hydration reaction for a period of 10–100 s. The CO_2_ concentrations ranged from 1.7 to 17 mM for the determination of the kinetic parameters and inhibition constants. Enzyme concentrations ranged between 5–12 nM. For each inhibitor, at least six traces of the initial 5–10% of the reaction were used to determine the initial velocity. The uncatalyzed rates were determined in the same manner and subtracted from the total observed rates. Stock solutions of the inhibitor (0.1 mM) were prepared in distilled–deionized water and dilutions up to 0.01 nM were done thereafter with the assay buffer. Inhibitor and enzyme solutions were preincubated together for 6 hrs at room temperature prior to the assay, to allow for the formation of the E–I complex. The inhibition constants were obtained by non-linear least-squares methods using PRISM 3 and the Cheng–Prusoff equation as reported earlier, and represent the mean from at least three different determinations. All CA isoforms were recombinant proteins obtained in-house, as reported earlier [18,19,20,21].

### 3.4. Preparation of Protein Structures

The crystal structures of hCA IX (PDB 3iai) and hCA XII (PDB 1jd0) co-crystallized with acetazolamide were obtained from the RCSB Protein Data Bank. Afterwards, the crystal structures of hCA II in complex with open-coumarin analogs were downloaded as well, i.e., in complex with 3-(4-methoxyphenyl)but-2-enoic acid (directly bound to the Zn^2+^ ion; PDB 5eh8), in complex with the open-coumarin 3-(2,4-dichlorophenyl)prop-2-enoic acid (interacts with the zinc-bound water molecule; PDB 5ehw), and in complex with (2*E*)-3-(2-hydroxyphenyl)acrylic acid (interacts with entrance amino acids of active site; PDB 5bnl). The structures were superposed on hCA IX and XII and the coordinates of the open-coumarin analog and the zinc-bound water molecule were copied into both hCA IX and XII. Subsequently, the structures were prepared using the protein preparation tool of Schrödinger (v2022-1, Schrödinger, Inc., New York, NY, USA). All water (except the zinc-bound water) and buffer molecules were omitted. Subunit A was retained and all other subunits, if present, were omitted. Subsequently, hydrogen atoms were added, and the system was minimized using the OPLS4 forcefield.

### 3.5. Docking Studies

Ligands **14a** and **22a** were prepared in the open and closed coumarin form using the LigPrep tool of Schrödinger and minimized with the OPLS4 forcefield. Subsequently, the ligands in the open-coumarin form were docked into the binding sites of the prepared protein structures. The binding sites were assigned as all residues within 5 Å of the co-crystallized ligands. Docking was performed using the Glide tool of Schrödinger with the SP settings. The pose generation was guided by the core of the co-crystallized open-coumarin analog. The closed-coumarin forms of the ligands were docked in the same way, however no core-guided docking was used. The three highest scoring poses were obtained for each ligand and the poses were subsequently minimized using the Prime tool and MM-GBSA forcefield. To this end, the docked ligand and all residues within 5 Å (with the exception of Zn^2+^, His94, His96 and His119) were unrestrained. High scoring compounds that formed binding interactions (hydrogen bonds, electrostatic interactions and hydrophobic interactions) and showed complementarity in shape and (a)polarity were selected for molecular dynamics (MD) simulations.

### 3.6. Molecular Dynamics Simulations

The ligand–enzyme complexes obtained with the docking procedure were subjected to a 250 ns MD simulation using Desmond. The complex was first placed in an orthorhombic box (at least 10 Å between complex and boundary) and then filled with Tip5P water molecules and 0.15 M NaCl. The amount of Na^+^ or Cl^−^ ions were adjusted to create a neutral system. Afterwards, all heavy atoms were restrained, and the system was minimized for 100 ps using the OPLS4 forcefield. Finally, the system was simulated for 250 ns under isothermic (Nose–Hoover chain, 1ps relaxation time) and isobaric (Martyna–Tobial–Klein, 2 ps relaxation time, isotropic coupling) conditions without restraints. Snapshots were saved every 250 ps. Finally, the percentage occurrence of the ligand–protein binding interactions as well as the MM-GBSA binding energy were calculated.

## 4. Conclusions

In conclusion, we reported a series of 6- and 7-substituted coumarins bearing variegated aryl thiosemicarbazide tails and we investigated their in vitro inhibition activity against the physiologically relevant hCA isoforms I, II, IX and XII. All compounds were effective inhibitors against the tumor-related isoforms IX and XII with K_i_ values spanning between the medium-low nanomolar range. The housekeeping hCAs I and II did not show appreciable inhibition, with the K_i_s > 10,000 nM. The binding modes of such compounds were explored by means of modelling experiments carried out on selected compounds assumed in their open form and in the thermodynamically most favored geometry (i.e., **14a-(*E*)-open** and **22a-(*E*)-open**). Molecular modelling suggests multiple interactions within the enzymatic cavity and may explain the high potency and selectivity reported for the hCAs IX and XII.

## Figures and Tables

**Table 1:** Inhibition data of hCA isoforms I, II, IX and XII with compounds **14a,b**–**22a,b** and **23b** and coumarin (**COU**) by the stopped flow CO_2_ hydrase assay [12].

Compound	hCA I (K_i_; nM *)	hCA II (K_i_; nM *))	hCA IX (K_i_; nM *))	hCA XII (K_i_; nM *))
**14a**	>10,000	>10,000	8.8	739
**14b**	>10,000	>10,000	46	743.5
**15a**	>10,000	>10,000	234	709
**15b**	>10,000	>10,000	78	684
**16a**	>10,000	>10,000	48	608
**16b**	>10,000	>10,000	55	866
**17a**	>10,000	>10,000	39	494
**17b**	>10,000	>10,000	64	745
**18a**	>10,000	>10,000	9.6	8.4
**18b**	>10,000	>10,000	721	7.5
**19a**	>10,000	>10,000	9.6	79
**19b**	>10,000	>10,000	9.4	573
**20a**	>10,000	>10,000	92	306
**20b**	>10,000	>10,000	70	56.6
**21a**	>10,000	>10,000	71	328.5
**21b**	>10,000	>10,000	9.1	8.6
**22a**	>10,000	>10,000	57	4
**22b**	>10,000	>10,000	33	4.6
**23b**	>10,000	>10,000	437.5	473
**COU [10]**	3100	9200	>10,000	>10,000

* Mean from 3 different assays, by the stopped flow technique (errors were in the range of ±5–10% of the reported values).

## Data Availability

Not applicable.
